# One year later: tracking the continued growth of mental illness stigma in England

**DOI:** 10.1192/bjo.2026.11050

**Published:** 2026-05-11

**Authors:** Amy Ronaldson, Claire Henderson

**Affiliations:** Health Services and Population Research Department, Institute of Psychiatry, Psychology & Neuroscience (IoPPN), King’s College London, UK

**Keywords:** Mental health, stigma and discrimination, Time to Change, social distance, population mental health

## Abstract

Using data from the Attitudes to Mental Illness (AMI) survey, we previously reported positive change in mental health stigma in England between 2008 and 2019. However, following the conclusion of the Time to Change campaign in 2021, 2023 data revealed a deterioration in several stigma-related attitudes. This report presents AMI survey 2024 results, examining changes over the past year. Regression analyses assessed stigma-related knowledge (Mental Health Knowledge Schedule (MAKS)), attitudes (Community Attitudes toward the Mentally Ill scale (CAMI)) and behavioural intent (Reported and Intended Behaviour Scale (RIBS-IB)), along with willingness to interact based on vignettes of depression and schizophrenia. The proportion of respondents achieving 2023-level MAKS and CAMI scores declined significantly (by 3.5% (*p* = 0.028) and 7.0% (*p* < 0.001), respectively), whereas RIBS-IB scores showed a non-significant decrease. Vignette responses remained stable, but there are signs of increasing desire for social distance. This report explores potential drivers of these trends.

Public stigma towards people with mental illness comprises the beliefs, attitudes and behaviour towards people perceived as having, or with a diagnosis of, a mental illness. We previously found evidence that public stigma in England worsened over the periods 1994–2003^1^ and 2019–2023.^2^ There was a significant reduction in public stigma between these periods, particularly in 2008 and 2019.^3^ The Time to Change stigma reduction programme^4^ ran from 2009 to 2021, including a social marketing campaign and work with several target groups, and may have contributed to this improvement.^5^

Other stigma measures from this period are available for comparison. Surveys of discrimination experienced by people using mental health services, undertaken between 2008 and 2014, showed a significant reduction overall, and in numerous areas of social life and employment.^6^ General population surveys measuring desire for social distance from people, described in vignettes with symptoms of depression and schizophrenia, also showed improvements between 2007 and 2021.^2^ This was maintained between 2021 and 2023,^2^ in contrast to the deterioration in public stigma measured with scales comprising items about people with mental illness in general.

This divergence suggests that interpersonal intentions towards known individuals should be distinguished from attitudes towards people with mental illness in general. To continue tracking general population stigma, we aimed to compare both aspects of stigma between 2023 and 2024.

## Method

Data from the Attitude to Mental Illness (AMI) survey are available from 2008. The latest survey ran from 16 September to 10 November 2024. A quota-sampling frame was used to ensure a nationally representative sample of adults (16 years or older) living in England; respondents were not resampled in later surveys, and approximately 1700 respondents took part each time. Information about the data source, sampling methods and study measures are published elsewhere^2^ and in the Supplementary Methods. The King’s College London Psychiatry, Nursing and Midwifery Research Ethics Subcommittee exempted analysis of these data as secondary analysis of anonymised data.

Measures of stigma-related knowledge (Mental Health Knowledge Schedule (MAKS^7^)), attitudes to mental illness (Community Attitudes toward the Mentally Ill (CAMI^8^)) and desire for social distance (Reported and Intended Behaviour Scale (RIBS-IB^9^)) were included, as they have been since 2008 (CAMI) and 2009 (MAKS, RIBS-IB). For the second time, we included vignettes from the British Social Attitudes Surveys^10^ depicting a common mental health problem (‘Stephen’: depression) and a less common problem (‘Andy’: schizophrenia). Participants were asked social distancing questions assessing their willingness to engage with the vignette character across six domains: living next door, socialising, forming a friendship, working as colleagues, accepting them as a family member by marriage and having them provide childcare.

In the current study, we compared 2024 outcomes with those from 2023 by estimating adjusted mean differences. These differences were then converted into changes in the proportion of individuals scoring above the 2023 mean, which served as the reference threshold. We used survey-weighted, multiple regression models to evaluate patterns of change in MAKS, CAMI (including CAMI subscales: ‘Prejudice and Exclusion’, ‘Tolerance and Support for Community Care’) and RIBS-IB scores. All models used the standardised scores of the measures as dependent variables, meaning that outputs were interpreted in standard deviation units. Logistic regression models were used to assess change in vignette responses (willing versus unwilling) between 2023 and 2024. Models were adjusted for sociodemographic variables: age, gender, ethnicity, socioeconomic position and government office region.^2^

Analyses were performed with Stata 18.0 (Stata Corp, College Station, Texas, USA).

## Results

Detailed characteristics for the samples up to 2023 were provided previously.^2^ The 2024 sample comprised 1563 participants with the distribution of gender and age group remaining stable, whereas changes since the introduction of remote data collection in 2019 (i.e. more participants with professional/managerial occupations, greater familiarity with mental health problems) were sustained.

**Fig. 1:**
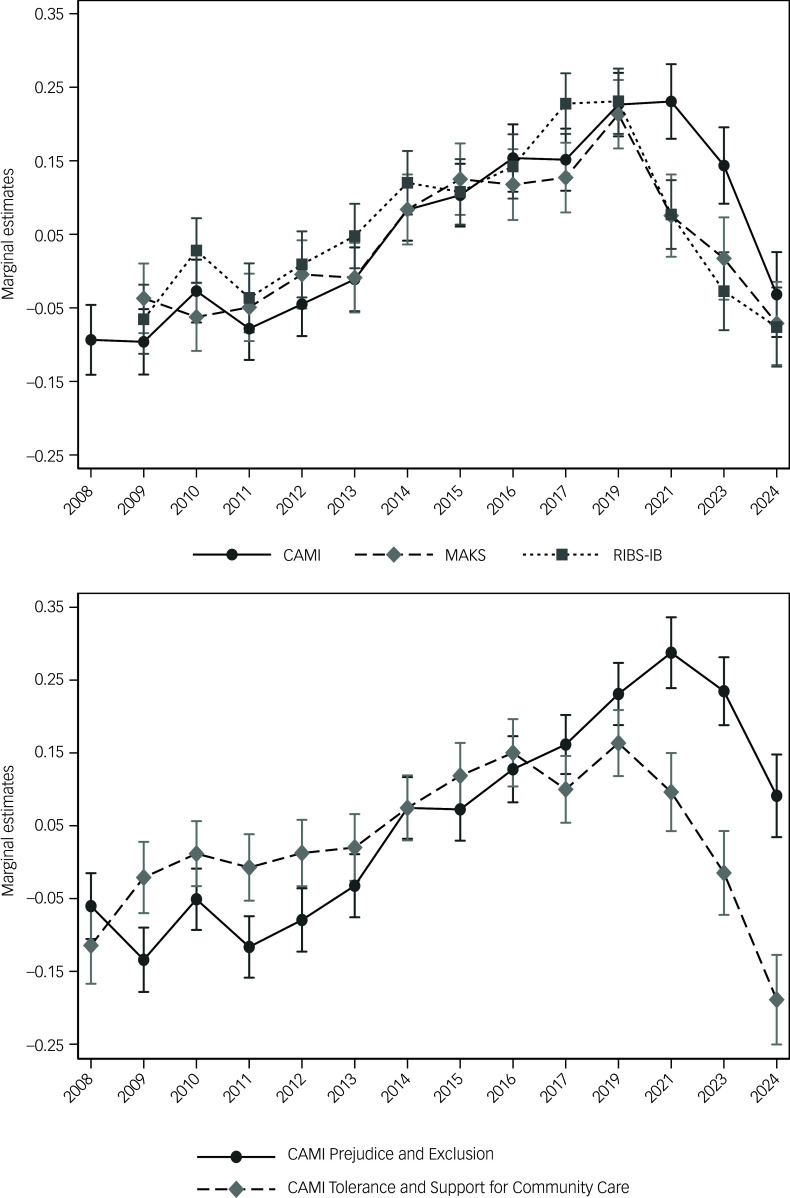
Top, marginal estimates of stigma-related attitudes (CAMI), knowledge (MAKS) and desire for social distance (RIBS-IB) by year (confidence intervals). Bottom, marginal estimates for subscales of the CAMI scale. RIBS-IB, Reported and Intended Behaviour Scale.

Survey-weighted descriptive statistics were calculated for all outcome measures for 2023 and 2024 (Supplementary Table 1). Figure 1 depicts change over time in total CAMI (plus subscales), MAKS and RIBS-IB scores. Previously we reported decreases in all scale scores between 2019 and 2023.^2^ Between 2023 and 2024, the proportion of respondents reaching 2023-level MAKS scores reduced significantly, by 3.5% (*p* = 0.028). The proportion of respondents reaching 2023-level total CAMI scores decreased by 7.0% (*p* < 0.001). Analysis of the CAMI subscales showed a 5.7% reduction in the proportion of respondents achieving 2023-level scores on the Prejudice and Exclusion subscale (*p* < 0.001), and a 6.9% reduction on the Tolerance and Support for Community Care subscale (*p* < 0.001). The proportion of respondents reaching 2023-level RIBS-IB scores declined by 1.9% but was not statistically significant (*p* = 0.202). Regression coefficients are presented in Supplementary Table 1.

Across vignettes, between 2023 and 2024 the proportion of people who were fairly or very unwilling to interact with ‘Andy’ (schizophrenia) increased across most scenarios, with the exception of working as colleagues and providing childcare. However, fully adjusted logistic regression models showed that none of the changes reached statistical significance. Fully adjusted models also revealed no statistically significant changes in unwillingness to interact with ‘Stephen’ (depression), although increases were observed in the proportion of people unwilling to interact across the following: socialising, forming a friendship, working as colleagues, accepting them as a family member by marriage and having them provide childcare. Descriptive statistics and results from regression models are provided in Supplementary Table 2.

## Discussion

Further deterioration in public stigma occurred over the period 2023–2024 regarding people with mental illness/mental health problems in general, although desire for social distancing from named individuals, conveying familiarity, has not shown significant change. Negative stereotypes, leading to stigmatisation in the context of unequal power relationships between people with mental ill health and others,^11^ may be easier to apply to groups of unknown people rather than towards an individual described as if they were known. However, it should not be assumed there will be no future increase in interpersonal stigma, just as it decreased previously in line with public stigma^3^ and experiences of discrimination.^6^ Despite study limitations (see Supplementary Material), there are grounds for concern that people with mental ill health will, or may already, experience greater active discrimination and/or avoidance by others.

Several factors may be contributing to increasing public stigma. Rising levels of mental ill health and help-seeking among young people have been interpreted by some public figures as evidence that they are ‘work-shy’ and misusing the welfare system.^12^ From a systems perspective,^13^ greater help-seeking and rising levels of mental ill health are disruptive, leading to either greater investment in services or a backlash, reflecting economic and labour market circumstances and/or a failure to address structural stigma. The use of vignettes of people with common mental disorder that vary by age and employment status could be used to explore the intersectionality of these characteristics. Homicides by people with psychosis that receive intense media coverage and criticism of the mental health services responsible for the care of the perpetrator are likely to influence public stigma.^14,15^ However, given long delays in hospitalisation in parts of England, community members’ anxiety about unwell people in their neighbourhood is increasingly likely. We suggest that this should not be dismissed as public stigma: for example, instead of a process that starts with labelling, fear may then be followed by recognition that someone is unwell and thence a general desire for social distancing from people with such illnesses. However, within socially deprived communities, enacting social distancing may not be possible and there may be relatively little power differential. We therefore recommend qualitative exploration of the experience and views of people in such communities.

## Supporting information

10.1192/bjo.2026.11050.sm001 Ronaldson and Henderson supplementary material Ronaldson and Henderson supplementary material

## Data Availability

The data that support the findings of this study are available from the corresponding author, A.R., upon reasonable request.

## Supplementary material

The supplementary material is available online at https://doi.org/10.1192/bjo.2026.11050

## References

[1] Mehta N , Kassam A , Leese M , Butler G , Thornicroft G. Public attitudes towards people with mental illness in England and Scotland, 1994–2003. Br J Psychiatry 2009; 194: 278–84. 19252160 10.1192/bjp.bp.108.052654

[2] Ronaldson A , Henderson C. Investigating changes in mental illness stigma and discrimination after the Time to Change programme in England. BJPsych Open 2024; 10: e199. 39501845 10.1192/bjo.2024.801 PMC11698152

[3] Henderson C , Potts L , Robinson EJ. Mental illness stigma after a decade of Time to Change England: inequalities as targets for further improvement. Eur J Public Health 2020; 30: 526–32. 32531039 10.1093/eurpub/ckaa013 PMC7292343

[4] Potts LC , Henderson C. Evaluation of anti-stigma social marketing campaigns in Ghana and Kenya: Time to Change Global. BMC Public Health 2021; 21: 886. 33964900 10.1186/s12889-021-10966-8 PMC8106856

[5] Evans-Lacko S , Corker E , Williams P , Henderson C , Thornicroft G. Effect of the Time to Change anti-stigma campaign on trends in mental-illness-related public stigma among the English population in 2003-13: an analysis of survey data. Lancet Psychiatry 2014; 1: 121–8. 26360575 10.1016/S2215-0366(14)70243-3

[6] Corker E , Hamilton S , Robinson E , Cotney J , Pinfold V , Rose D , et al. Viewpoint survey of mental health service users’ experiences of discrimination in England 2008-2014. Acta Psychiatr Scand 2016; 134: 6–13. 10.1111/acps.12610 PMC6681145 27426641

[7] Evans-Lacko S , Little K , Meltzer H , Rose D , Rhydderch D , Henderson C , et al. Development and psychometric properties of the Mental Health Knowledge Schedule. Can J Psychiatry 2010; 55: 440–8. 20704771 10.1177/070674371005500707

[8] Taylor SM , Dear MJ. Scaling community attitudes toward the mentally ill. Schizophr Bull 1981; 7: 225–40. 7280561 10.1093/schbul/7.2.225

[9] Evans-Lacko S , Rose D , Little K , Flach C , Rhydderch D , Henderson C , et al. Development and psychometric properties of the reported and intended behaviour scale (RIBS): a stigma-related behaviour measure. Epidemiol Psychiatr Sci 2011; 20: 263–71. 21922969 10.1017/s2045796011000308

[10] NatCen Social Research. British Social Attitudes Survey, 2015. NatCen Social Research, 2017.

[11] Link BG , Yang LH , Phelan JC , Collins PY. Measuring mental illness stigma. Schizophr Bull 2004; 30: 511–41. 15631243 10.1093/oxfordjournals.schbul.a007098

[12] Weaver M. Jeremy Hunt and Mel Stride Warn Against Benefits ‘Lifestyle Choice’ . The Guardian, 2024 (https://www.theguardian.com/society/article/2024/may/15/jeremy-hunt-mel-stride-warn-benefits-lifestyle-choice-unemployment).

[13] Skivington K , Matthews L , Simpson SA , Craig P , Baird J , Blazeby JM , et al. A new framework for developing and evaluating complex interventions: update of Medical Research Council guidance. BMJ 2021; 374: n2061. 34593508 10.1136/bmj.n2061 PMC8482308

[14] Corrigan PW , Powell KJ , Michaels PJ. The effects of news stories on the stigma of mental illness. J Nerv Ment Dis 2013; 201: 179–82. 23407209 10.1097/NMD.0b013e3182848c24

[15] Cummins I. Psychiatry, racism and crime: the case of Christopher Clunis reconsidered. Front Psychiatry 2024; 15: 1334020. 38384593 10.3389/fpsyt.2024.1334020 PMC10880558

