# Supplementary material for: One year later: tracking the continued growth of mental illness stigma in England
Source: BJPsych Open. 2026 May 11;12(3):e135. doi: 10.1192/bjo.2026.11050 (PMC13169051; doi:10.1192/bjo.2026.11050)
Supplement: Ronaldson and Henderson supplementary material [file S2056472426110503sup001.docx]

**One year later: Tracking the continued growth of mental illness stigma in England**

**Supplementary Material**

**Supplementary Methods**

*Data source and sampling*

The AMI has been carried out in England annually from 2008 to 2017, and every 2 years since 2017 by Verian (previously Kantar), with the latest in 2024. Approximately 1700 respondents take part at each wave. A quota sampling frame is used to ensure a nationally representative sample of adults (16 years or older) living in England who could be exposed to the Time to Change campaign, and respondents are not resampled in later surveys. Detailed information about sampling methods can be obtained via the authors. Until 2019, respondents were interviewed face to face at home, by trained personnel. Since then, data have been collected using address-based online surveying (<https://www.kantar.com/-/media/Project/Kantar/Global/Expertise/Policy-and-Society/Address-Based-Online-Surveying.PDF>), which offers web or paper self-completion.

*Measures*

Measures included a scale measuring stigma-related knowledge (Mental Health Knowledge Schedule (MAKS)), a scale measuring attitudes to mental illness (Community Attitudes toward the Mentally Ill (CAMI)), and a scale measuring desire for social distance (Reported and Intended Behaviour Scale) (RIBS)). Desire for social distance was further explored using vignettes portraying depression and schizophrenia.

Stigma-related knowledge was measured using the first six items of the Mental Health Knowledge Schedule (MAKS)^1^, covering help-seeking, recognition, support, employment, treatment and recovery (e.g. ‘Most people with mental health problems want to have paid employment’). The standardised total score was used. These items were rated on a scale ranging from 1 (strong disagreement) to 5 (strong agreement). Scores can range from 6 to 30, with higher scores indicating greater knowledge. The Cronbach's alpha for the total scale in the current study was 0.59. This relatively low internal consistency has been reported previously^2,3^, and likely reflects the different knowledge domains measured by each item^1^.

We used 26 of the 40 items of the CAMI scale^4^ in the current study plus an item on employment-related attitudes added when the survey was first commissioned by the UK Department of Health. The CAMI has two factors: ‘Prejudice and Exclusion’ (e.g. ‘One of the main causes of mental illness is a lack of self- discipline and willpower’) and ‘Tolerance and Support for Community Care’ (e.g. ‘We need to adopt a far more tolerant attitude toward people with mental illness in our society’). All items are rated on a scale ranging from 1 (strong disagreement) to 5 (strong agreement), and scores can range from 26 to 130. The standardised scores of the total CAMI, ‘Prejudice and Exclusion’ and ‘Tolerance and Support for Community Care’ subscales were used. Higher scores indicate less stigmatising attitudes to mental illness. The internal consistency of the CAMI in 2024 was 0.89 (Cronbach’s alpha) for the total scale, and 0.87 and 0.79 for ‘Prejudice and Exclusion’ and ‘Tolerance and Support for Community Care’, respectively.

Desire for social distance was measured with the four items of the Reported and Intended Behaviour Scale (RIBS)^5^, which measure intended behaviour. These four items assess desire for social distance in terms of living with, working with, living nearby and continuing a relationship with someone with a mental illness. These items were rated on a scale ranging from 1 (strong disagreement) to 5 (strong agreement). Scores can range from 4 to 20, with higher scores indicating less desire for social distance. The total score was standardised. The internal consistency of the four items was 0.85 (Cronbach's alpha).

We included vignettes of a common mental health problem (depression) and a less common problem (schizophrenia). Two different people were described, and respondents were asked how willing they would be to interact with them in a range of situations (e.g. spend time socialising, move next door to). The vignettes were not labelled ‘depression’ or ‘schizophrenia’. People were asked social distance questions for each vignette about willingness to have the person in the vignette as a neighbour, to socialise with them, to have them care for their children, to befriend them, to work with them and to have them marry someone they know. Participants responded to these items on a five-point Likert type scale, with 1 being ‘very willing’, 2 being ‘fairly willing’, 3 being ‘neither willing nor unwilling’, 4 being ‘fairly unwilling’ and 5 being ‘very unwilling’. Higher scores indicate more desire for social distance. In the current study we compared AMI vignettes from 2023 and 2024.

*Covariates*

Covariates included age at completion of the survey, self-reported gender, self-reported ethnicity (Asian, Black, Other, White), socioeconomic position and government office region. Socioeconomic position was based on the chief income earner of each household, using the Market Research Society's classification system (AB, C1, C2, DE). AB represents professional /managerial occupations, C1 represents other non-manual occupations, C2 represents skilled manual occupations, and DE represents semi-/unskilled manual occupations and people dependent on state benefits. Government office region is the lowest level information on participants’ location as described by the UK Government's Office for National Statistics (ONS).

**Supplementary Results**

| **Table S1**. Survey-weighted descriptive statistics and results from adjusted linear regressions for MAKS, CAMI, and RIBS-IB scales in 2023 (N=1638) and 2024 (N=1563) | | | | | | |
| --- | --- | --- | --- | --- | --- | --- |
| Outcome | Year | Weighted mean | SD | 95% CI | Skewness | β (95% CI) |
| MAKS | 2023 | 23.0 | 3.2 | 22.8 to 23.2 | -0.25 | Reference |
| MAKS | 2024 | 22.7 | 3.2 | 22.5 to 22.9 | -0.21 | -0.09 (-0.17 to -0.01)* |
| CAMI | 2023 | 111.3 | 13.9 | 110.5 to 112.1 | -0.77 | Reference |
| CAMI | 2024 | 108.8 | 14.8 | 108.0 to 109.6 | -0.71 | -0.17 (-0.25 to -0.10)** |
| RIBS-IB | 2023 | 16.5 | 3.5 | 16.3 to 16.7 | -1.03 | Reference |
| RIBS-IB | 2024 | 16.3 | 3.4 | 16.1 to 16.5 | -0.88 | -0.05 (-0.12 to 0.03) |
| Regression results are based on standardised scores from outcome measures  *p<0.05; **p<0.001  CAMI= Community Attitudes toward the Mentally Ill; CI=confidence interval; MAKS= Mental Health Knowledge Schedule; RIBS-IB= Reported and Intended Behaviour Scale – Intended Behaviour; SD=standard deviation | | | | | | |

| **Table S2.** Descriptive statistics for schizophrenia and depression vignettes and results from logistic regression models (reference=2023) | | | | |
| --- | --- | --- | --- | --- |
|  | **2023 AMI** | **2024 AMI** |  |  |
| *Vignette: Schizophrenia* | *% of people who were fairly or very unwilling* | *% of people who were fairly or very unwilling* | *Unadjusted OR (95% CI)** | *Adjusted OR*  *(95% CI)** |
| \| And now we would like you to think about how willing, or unwilling, you would be to ... \| \| --- \| |  |  |  |  |
| *…move next door to Andy/Gareth?* | 299 (20.2) | 289 (20.5) | 1.02 (0.85 to 1.22) | 1.03 (0.85 to 1.25) |
| *…spend time socializing with Andy/Gareth?* | 269 (17.9) | 261 (18.5) | 1.04 (0.86 to 1.25) | 1.02 (0.83 to 1.24) |
| *…make friends with Andy/Gareth?* | 230 (15.1) | 235 (16.3) | 1.10 (0.90 to 1.33) | 1.10 (0.89 to 1.36) |
| *…have Andy/Gareth as a colleague/workmate?* | 210 (14.0) | 198 (13.7) | 0.98 (0.79 to 1.20) | 1.05 (0.84 to 1.32) |
| *…have Andy/Gareth marry into the family?* | 452 (32.5) | 442 (33.1) | 1.03 (0.88 to 1.21) | 1.08 (0.91 to 1.29) |
| *…have Andy/Gareth provide childcare for someone in your family?* | 909 (65.4) | 852 (63.2) | 0.91 (0.78 to 1.06) | 0.94 (0.79 to 1.11) |
|  |  |  |  |  |
| *Vignette: Depression* | *% of people who were fairly or very unwilling* | *% of people who were fairly or very unwilling* | *Unadjusted OR (95% CI)** | *Adjusted OR*  *(95% CI)** |
| \| And now we would like you to think about how willing, or unwilling, you would be to ... \| \| --- \| |  |  |  |  |
| *…move next door to Stephen?* | 84 (5.5) | 73 (5.3) | 0.97 (0.71 to 1.34) | 1.02 (0.72 to 1.43) |
| *…spend time socializing with Stephen?* | 105 (6.8) | 123 (8.3) | 1.25 (0.95 to 1.64) | 1.22 (0.90 to 1.63) |
| *…make friends with Stephen?* | 106 (6.8) | 111 (7.5) | 1.11 (0.84 to 1.46) | 1.04 (0.76 to 1.40) |
| *…have Stephen as a colleague/workmate?* | 109 (7.2) | 124 (8.5) | 1.20 (0.92 to 1.57) | 1.20 (0.90 to 1.60) |
| *…have Stephen marry into the family?* | 307 (21.3) | 282 (20.6) | 0.96 (0.80 to 1.15) | 0.98 (0.80 to 1.20) |
| *…have Stephen provide childcare for someone in your family?* | 592 (41.6) | 572 (42.2) | 1.02 (0.88 to 1.19) | 1.10 (0.94 to 1.30) |
| *Logistic regression models assessed change in vignette responses (willing versus unwilling) between 2023 and 2024. 2023 acted as the reference year.  AMI=Attitudes to Mental Illness; CI=confidence interval; OR=odds ratio | | | | |

**Study limitations**

There are a number of study limitations to be considered. To date, no epidemiological survey has allowed repeated assessments of mental health stigma. Therefore, although a quota sample was used in the current study, we calculated sampling errors to provide approximate indicators of uncertainty, while recognising that this approach does not fully meet the assumptions of probability sampling. Although we provide several plausible societal explanations for the increase in mental health stigma we observed, we acknowledge that these are speculative and causality cannot be supported by the data.  Although we included a significant number of confounders in our analyses, it is possible there were unmeasured confounders that may bias our results. A change in data collection method (i.e. face to face up to 2019, followed by paper- and web-based completion since 2021) may partially explain increases in stigma seen from 2021 resulting from reduced likelihood of socially desirable responses. However, the change reported here is between 2023 and 2024, which is likely to be less affected by this issue. As with all self‑report measures, responses in this study may be influenced by social desirability and other reporting biases, which should be considered when interpreting the findings. A further limitation concerns the use of mental health vignettes, which may not fully represent real-world scenarios. However, these vignettes were based on established Diagnostic and Statistical Manual of Mental Disorders (DSM) criteria, and therefore accurately reflect the main symptoms of the mental health conditions they depict.

**References**

1 Evans-Lacko S, Little K, Meltzer H, Rose D, Rhydderch D, Henderson C, *et al.* Development and psychometric properties of the Mental Health Knowledge Schedule. *Can J Psychiatry* 2010; **55**: 440–8.

2 Henderson C, Potts L, Robinson EJ. Mental illness stigma after a decade of Time to Change England: inequalities as targets for further improvement. *Eur J Public Health* 2020; **30**: 526–32.

3 Ronaldson A, Henderson C. Investigating changes in mental illness stigma and discrimination after the Time to Change programme in England. *BJPsych Open* 2024; **10**: e199.

4 Taylor SM, Dear MJ. Scaling Community Attitudes Toward the Mentally Ill. *Schizophrenia Bulletin* 1981; **7**: 225–40.

5 Evans-Lacko S, Rose D, Little K, Flach C, Rhydderch D, Henderson C, *et al.* Development and psychometric properties of the reported and intended behaviour scale (RIBS): a stigma-related behaviour measure. *Epidemiol Psychiatr Sci* 2011; **20**: 263–71.
